# Protocol for a Process Evaluation of the Quality Improvement Intervention to Enhance Access to Kidney Transplantation and Living Kidney Donation (EnAKT LKD) Cluster-Randomized Clinical Trial

**DOI:** 10.1177/20543581221084502

**Published:** 2022-03-19

**Authors:** Seychelle Yohanna, Mackenzie Wilson, Kyla L. Naylor, Amit X. Garg, Jessica M. Sontrop, Dmitri Belenko, Lori Elliott, Susan McKenzie, Sara Macanovic, Istvan Mucsi, Rachel Patzer, Irina Voronin, Iris Lui, Peter G. Blake, Amy D. Waterman, Darin Treleaven, Justin Presseau

**Affiliations:** 1 Division of Nephrology, McMaster University, Hamilton ON, Canada; 2 St. Joseph’s Healthcare Hamilton, ON, Canada; 3 Clinical Epidemiology Program, Ottawa Hospital Research Institute, ON, Canada; 4 Department of Epidemiology & Biostatistics, Western University, London, ON, Canada; 5 Division of Nephrology, Western University, London, ON, Canada; 6 Ontario Renal Network, Ontario Health, Toronto, Canada; 7 Division of Nephrology, University of Toronto, ON, Canada; 8 Grand River Hospital, Kitchener, ON, Canada; 9 Health Services Research Center, School of Medicine, Emory University, Atlanta, USA; 10 Division of Nephrology, University of California, Los Angeles, USA; 11 Trillium Gift of Life Network, Toronto, ON, Canada

**Keywords:** process evaluation, theoretical domains framework, normalization process theory, fidelity, mechanisms of change, chronic kidney disease, kidney transplantation, living kidney donation, protocol

## Abstract

**Background:**

Many patients who would benefit from a kidney transplant never receive one. The Enhance Access to Kidney Transplantation and Living Kidney Donation (EnAKT LKD) pragmatic, cluster-randomized clinical trial is testing whether a multi-component quality improvement intervention, provided in chronic kidney disease (CKD) programs (vs. usual care), can help patients with CKD with no recorded contraindications to kidney transplant complete more steps toward receiving a transplant (primary outcome of the trial). The EnAKT LKD intervention has 4 components: (1) quality Improvement teams and administrative support, (2) improved transplant education for patients and healthcare providers, (3) access to support and (4) program-level performance monitoring.

**Objective:**

To conduct a process evaluation of the EnAKT LKD quality improvement intervention to determine if the components were delivered, received, and enacted as designed (fidelity), and if the intervention addressed intended barriers (mechanisms of change).

**Design:**

A mixed-methods process evaluation informed by new practice implementation and theories of behavior change.

**Setting:**

Chronic kidney disease programs in Ontario, Canada, began receiving the EnAKT LKD intervention on November 1, 2017 and will continue to receive it until December 31, 2021. The process evaluation (interviews and surveys) will occur alongside the trial, between December 2020 to May 2021.

**Participants:**

Healthcare providers (eg, dialysis nurses, nephrologists, members of the multi-care kidney clinic team) at Ontario’s 27 CKD programs.

**Methods:**

We will survey and interview healthcare providers at each CKD program, and complete an intervention implementation checklist. Quantitative data from the surveys and the intervention implementation checklist will assess fidelity to the intervention, while quantitative and qualitative data from surveys and interviews will provide insight into the mechanisms of change.

**Limitations:**

The long trial period may result in poor participant recall.

**Conclusion:**

This process evaluation will enhance interpretation of the trial findings, guide improvements in the intervention components, and inform future implementation.

**Trial registration:**

Clinicaltrials.gov; identifier: NCT03329521.

## Abrégé

**Contexte:**

Plusieurs patients qui pourraient tirer profit d’une greffe de rein n’en reçoivent jamais une. L’essai clinique pragmatique et randomisé par grappes EnAKT LKD (*Enhance Access to Kidney Transplantation and Living Kidney Donation*) vise l’amélioration de l’accès à la transplantation rénale et au don de rein vivant. L’essai examine une intervention d’amélioration de la qualité (par rapport aux soins habituels) à composantes multiples réalisée dans le cadre des programs d’insuffisance rénale chronique (IRC) afin de déterminer si elle peut aider les patients atteints d’une néphropathie chronique sans contre-indications documentées à une greffe rénale à franchir davantage d’étapes vers la réception d’une greffe (principal critère d’évaluation de l’essai). L’intervention EAKT LKD comporte quatre composantes : 1) les équipes d’amélioration de la qualité et le soutien administratif; 2) l’amélioration de l’éducation sur la transplantation destinée aux patients et aux fournisseurs de soins; 3) l’accès au soutien; et 4) le suivi du rendement à l’échelle du program.

**Objectif:**

L’évaluation du processus de l’intervention d’amélioration de la qualité EnAKT LKD vise deux objectifs : déterminer si les composants ont été livrés, reçus et mis en œuvre comme prévu (fidélité) et vérifier si l’intervention a permis d’éliminer les obstacles prévus (mécanismes de changement).

**Type d’étude:**

Une évaluation de processus à méthodes mixtes fondée sur les théories concernant la mise en œuvre de nouvelles pratiques et les changements de comportement.

**Cadre:**

Les programs d’IRC ontariens (Canada) ont commencé à recevoir l’intervention EnAKT LKD le 1er novembre 2017 et ont continué de la recevoir jusqu’au 31 décembre 2021. L’évaluation du processus (sondages et entretiens) s’est effectuée parallèlement à l’essai, de décembre 2020 à mai 2021.

**Participants:**

Les fournisseurs de soins (infirmières en dialyze, néphrologues, membres du personnel des cliniques multidisciplinaires en santé rénale) des 27 programs d’IRC ontariens.

**Méthodologie:**

Nous allons sonder et interroger les fournisseurs de soins de chaque program d’IRC et nous complèterons une liste vérifiant la mise en œuvre de l’intervention. Les données quantitatives tirées des sondages et listes de vérification permettront d’évaluer la fidélité à l’intervention, alors que les données quantitatives et qualitatives extraites des sondages et des entretiens fourniront un aperçu des mécanismes de changement.

**Limites:**

La longue période de l’essai pourrait rendre difficile le rappel des participants.

**Conclusion:**

Cette évaluation du processus permettra d’améliorer l’interprétation des résultats de l’essai et de guider l’amélioration des composantes de l’intervention, en plus d’éclairer de futures mises en œuvre.

**Enregistrement de l’essai:**

ClinicalTrials.gov; identifiant : NCT03329521

## Background

Kidney transplantation is the optimal treatment for many patients with kidney failure. Kidney transplantation compared to dialysis is associated with better patient survival, higher quality of life, and lower healthcare costs.^1-3^ Unfortunately, the number of patients in need of a transplant continues to grow.^4^ There is an insufficient number of deceased donors to meet this demand and the rate of living donor kidney transplantation has stagnated for several years.^5-7^ In addition to the shortage of transplantable kidneys, there are other barriers to transplantation, including a lack of knowledge about transplantation and living kidney donation, poor communication between chronic kidney disease (CKD) programs and transplant centers, and inefficiencies within the healthcare system.^8-10^

### The Enhance Access to Kidney Transplantation and Living Kidney Donation (EnAKT LKD) Trial

To improve access to kidney transplantation and living kidney donation in Ontario, Canada, a partnership was formed between 2 major provincial government-funded organizations: The Ontario Renal Network (ORN, part of Ontario Health), which manages the delivery of renal services in Ontario and the Trillium Gift of Life Network (TGLN, part of Ontario Health), which coordinates organ donation and transplant care in Ontario. This partnership resulted in the development of a multi-component quality improvement intervention: The Enhance Access to Kidney Transplantation and Living Kidney Donation (EnAKT LKD) intervention.^11^ As described elsewhere, this intervention is being tested in a cluster-randomized controlled trial. The overall objective of this trial is to determine if a quality improvement intervention provided in CKD programs (vs. usual care) enables more patients with no recorded contraindications to kidney transplant complete more steps toward receiving a kidney transplant.

In brief, the intervention includes 4 components:

1. Quality improvement teams and administrative support: Each CKD program established a local quality improvement team that received training, administrative support, and resources to implement the intervention.
2. Improved transplant education for patients and healthcare providers: Tailored educational resources were developed and delivered to CKD program staff, patients and living kidney donor candidates.
3. Access to support: The Transplant Ambassador Program (TAP: www.transplantambassadors.ca) connects patients with kidney disease and their families to kidney transplant recipients and living kidney donors who can share their lived experience with transplantation and living kidney donation.
4. Program-level performance monitoring: Ongoing data collection and reports are provided to CKD programs about their transplant-related performance using an audit-and-feedback approach.

Further details on the quality improvement intervention can be found in the main trial protocol^11^ and are elaborated in the Template for Intervention Description and Replication (TIDieR) checklist in Supplemental Appendix 1).^12^

### The EnAKT LKD Cluster-Randomized Clinical Trial

The EnAKT LKD trial is a 2-arm, parallel-group, open-label, registry-based, cluster-randomized clinical trial being conducted in all of Ontario’s 27 CKD programs. The primary outcome of the trial is the number of predefined steps that patients with CKD with no recorded contraindications to kidney transplant complete toward receiving a kidney transplant (see Figure 2). Thirteen of Ontario’s 27 CKD programs were randomly allocated to receive the intervention starting on November 1, 2017 (26 CKD programs existed in November 2017; 1 program was divided into 2 in April 2018 but continues to be considered as 1 program for the main trial). The original trial end date, planned for March 31, 2021, was extended to December 31, 2021 due to the COVID-19 pandemic, when on March 16, 2020 nearly all kidney transplants and evaluations for deceased and living donor transplants in Ontario were temporarily suspended.

### Usual Care

CKD programs randomized to usual care are continuing to provide support in accessing kidney transplants as usual and will receive the intervention after the trial ends.

### The Need for a Process Evaluation of the EnAKT LKD Trial

While the trial results will provide information on the effectiveness of the intervention, a process evaluation will provide insight into whether the intervention was delivered, received, and enacted as designed (fidelity), and if the intervention as delivered addressed the barriers it intended to address (mechanism of change).^13^ The EnAKT LKD trial requires multiple healthcare providers to modify a variety of activities. The intervention has several interacting components that target patients, healthcare providers, and administrative processes, and its implementation is supported by quality improvement teams in each CKD program. The teams have flexibility in how they implement each component; for example, they can tailor a component to function better in their local environment. Process evaluations are especially useful for evaluating pragmatic trials conducted at multiple sites with local adaptations in delivery.^13^ We will use this process evaluation to help us interpret the trial findings.

**Figure 1.**
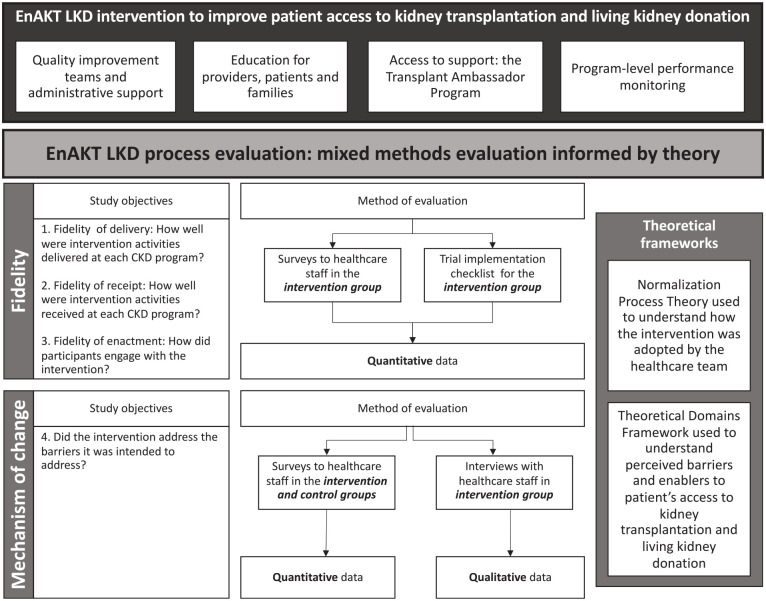
Design of the EnAKT LKD process evaluation. *Note.* EnAKT LKD = Enhance Access to Kidney Transplantation and Living Kidney Donation.

We will follow guidelines from the UK Medical Research Council process evaluation framework on the evaluation of complex interventions^14^ and use a mixed-methods approach. Figure 1 provides an overview of the process evaluation including the study objectives and design.

**Figure 2.**
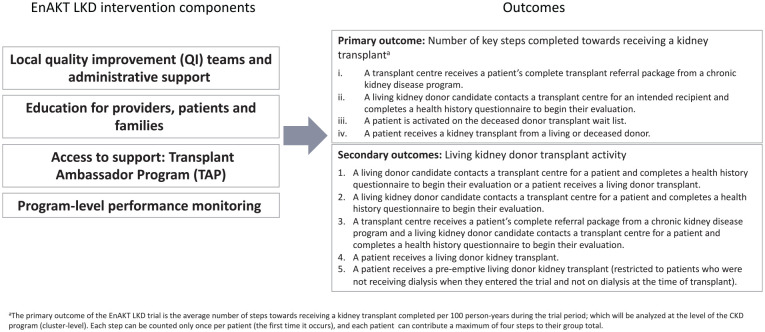
Overall EnAKT LKD intervention logic model. *Note.* EnAKT LKD = Enhance Access to Kidney Transplantation and Living Kidney Donation.

**Figure 3.**
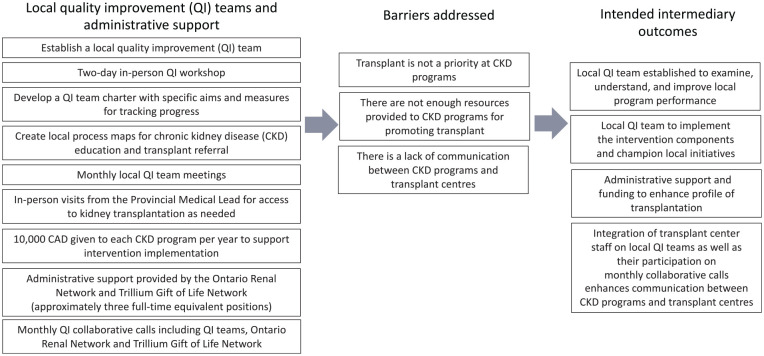
EnAKT LKD intervention component 1 logic model: Local quality improvement teams and administrative support. *Note.* EnAKT LKD = Enhance Access to Kidney Transplantation and Living Kidney Donation.

**Figure 4.**
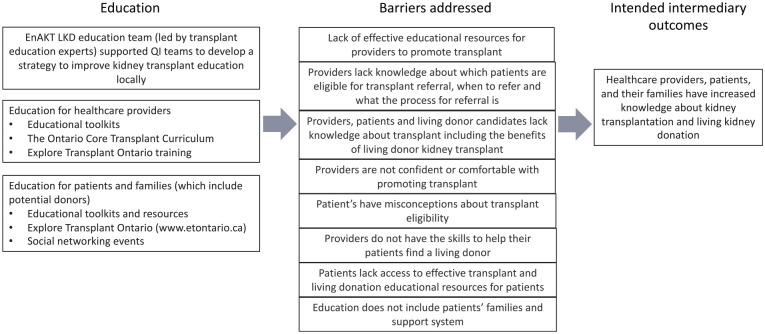
EnAKT LKD intervention component 2 logic model: Education for providers, patients, and families. *Note.* EnAKT LKD = Enhance Access to Kidney Transplantation and Living Kidney Donation.

**Figure 5.**
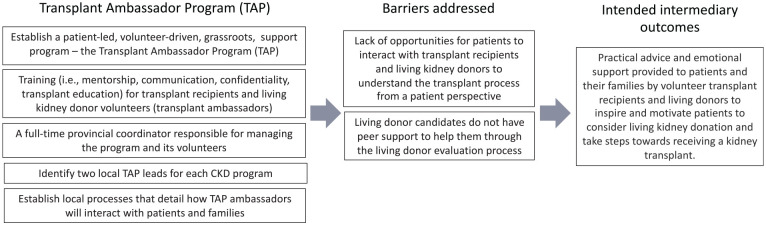
EnAKT LKD intervention component 3 logic model: Access to support—The Transplant Ambassador Program. *Note.* EnAKT LKD = Enhance Access to Kidney Transplantation and Living Kidney Donation.

**Figure 6.**
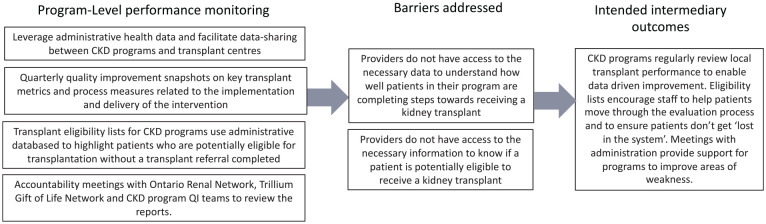
EnAKT LKD intervention component 4 logic model: Program-level performance monitoring. *Note.* EnAKT LKD = Enhance Access to Kidney Transplantation and Living Kidney Donation.

In consultation with the EnAKT LKD trial team, we developed a logic model to articulate assumptions about how each intervention component will produce an effect (Figures 2-6).^11^ A logic model depicts an intervention’s proposed causal pathways of change.

In this protocol, we define the objectives of our process evaluation, outline the methods we will use, and describe how we will integrate process and outcome data to analyze trial results. The results of our evaluation will enhance interpretation of the trial findings, guide improvements in the intervention components, and inform future implementation, including use of the intervention in the CKD programs in the usual-care arm in Ontario. In addition, our findings will contribute to the science of how best to translate interventions to increase access to kidney transplantation and living kidney donation into real-world practice.

## Objectives

The objectives of this process evaluation are to assess the following:

1. Fidelity of delivery: How well were intervention activities delivered at each CKD program?
2. Fidelity of receipt: How well were intervention activities received at each CKD program?
3. Fidelity of enactment: How did participants engage with the intervention?
4. Mechanisms of change: Did the intervention address the barriers it was intended to address?

## Methods

### Design

We will use mixed methods including an online survey, semi-structured interviews, and a trial implementation checklist to better understand how and why the EnAKT LKD intervention was effective or ineffective at increasing patients’ access to kidney transplantation. We will also assess potential control-group contamination through an online survey.

### Participants

*Surveys*: From December 2020 to February 2021, 6 healthcare providers from each of Ontario’s 27 CKD programs (13 programs in the intervention group and 13 in the control group) were invited to participate in an online survey. For each CKD program in the intervention group, the regional medical lead and the quality improvement team lead received the initial communication about the online survey. In the control group, the regional medical lead and the administrative lead received the initial communication about the online survey. They were asked to invite and recruit 6 healthcare providers from each CKD program, including 2 nephrologists, 2 dialysis nurses, and 2 multi-care kidney clinic team members (target N = 164 staff). The names of individuals invited to complete the online survey were not shared with the study team. Potential participants received an email inviting them to complete the survey with a link to the informed consent form and the survey. We followed-up with the quality improvement team leads on 2 occasions in 2- to 3-week intervals reminding them to invite participants to complete the survey.^15^

*Semi-structured interviews*: At the 13 CKD programs receiving the intervention, we also conducted one-on-one semi-structured virtual interviews with the leads of the quality improvement teams and one other healthcare professional (26 interviews total).

### Approach

In brief, the EnAKT LKD intervention components were designed to address barriers to transplantation. Further details about how the intervention components were developed are provided in the EnAKT LKD trial protocol.^11^ In this process evaluation, we will collect data to assess the quality of the intervention implementation (fidelity) and to assess if and how the intervention helped address barriers to kidney transplantation and living kidney donation (mechanism of change). The fidelity analysis will assess whether the components of the EnAKT LKD strategy were delivered, received, and enacted as designed (objectives 1-3). The measures used to assess each aspect of fidelity are described in Table 1. We mapped potential mechanisms of change (objective 4) onto 2 theoretical frameworks (see Supplemental Appendix IV) used to understand new practice implementation and behavioral change in healthcare settings (Normalization Process Theory and Theoretical Domains Framework). Normalization Process Theory (NPT) describes how new healthcare activities become routine and embedded over time in healthcare teams.^16^ NPT focuses on what people do rather than what they believe or intend. NPT captures information on 4 constructs: (a) how people make sense of change, (b) how they engage with change, (c) the operational work done to enact change, and (d) how they appraise how change is impacting themselves and others. The second framework used is the Theoretical Domains Framework (TDF), which synthesizes over 30 theories of behavior change into 14 domains that can be used as categories of barriers and enablers that are targeted by an intervention to assess whether the intervention affected the barriers it was designed to address.^17,18^ These complementary theoretical frameworks are embedded within the survey and interview guide to help understand potential mechanisms of change associated with the EnAKT LKD intervention.

**Table 1.** Fidelity of Intervention Delivery, Receipt, and Enactment Indicators, by Intervention **Component**.

EnAKT LKD intervention component	Fidelity of delivery indicators	Fidelity of receipt indicators	Fidelity of enactment indicators
**1. Quality improvement teams and administrative support**			
	• Confirmation of funds transferred to CKD programs for implementation of the strategy^a^ • Formal letter sent to CKD program administrators and directors to introduce the strategy^a^ • ‘How to create a QI team’ manual shared^a^ • Advanced QI training (IDEAS program) held^a^ • Number of in-person collaborative meetings held^a^ • Number of monthly collaborative calls held^a^ • Number of site visits by the Provincial Medical Lead^a^	• QI team members identified^a^ • Frequency of *planned* QI team meetings prior to the COVID-19 pandemic (prior to March 2020)^b^ • Frequency of QI team meetings *held* prior to the COVID-19 pandemic (prior to March 2020)^b^ • Frequency of *planned* QI team meetings during the first wave of the COVID-19 pandemic (March—May 2020)^b^ • Frequency of QI team meetings *held* during the first wave of the COVID-19 pandemic (March—May 2020)^b^ • Program charter with specific aims developed^a^ • Process map of the transplant referral pathway developed^a^ • Process map of the transplant education pathway developed^a^ • Proportion of survey respondents who . . . • took part in creating a QI team charter with specific aims^b^ • thought creating a program charter was helpful^b^ ○ took part in mapping the transplant referral process^b^ ○ thought mapping the transplant referral process was helpful^b^ ○ took part in mapping the transplant education pathway^b^ ○ thought mapping the transplant education pathway was helpful^b^ • Attendance at advanced QI training (IDEAS program, https://www.ideasontario.ca/)^a^ • Proportion of in-person collaborative meetings attended by at least one CKD program representative^a^ • Proportion of monthly collaborative calls attended by at least one CKD program representative^a^	• Number of members on the QI team^a^ • Mean number of QI team meetings held per quarter^a^ • Mean proportion of QI team members in attendance at QI team meetings^a^ • Proportion of survey respondents attending . . . ○ QI team meetings prior to the COVID-19 pandemic (prior to March 2020)^b^ ○ QI team meetings during the first wave of the COVID-19 pandemic (March—May 2020)^b^ • Number of program charter updates^a^ • Number of Plan-Do-Study-Act (PDSA) cycles conducted^a^ • Average number of Plan-Do-Study-Act cycles that survey respondents were involved with in an average quarter prior to the COVID-19 pandemic (prior to March 2020)^b^ • Average number of Plan-Do-Study-Act cycles that survey respondents were involved with in an average quarter during the first wave of the COVID-19 pandemic (March—May 2020)^b^
**2. Improved transplant education for patients and healthcare providers**			
Education for patients, families, and potential donors	• Number of Explore Transplant Ontario packages distributed per patient in the program since the EnAKT LKD trial outset^a^	• Number of patients who accessed the Explore Transplant Ontario website^a^ • Mean time (minutes) and SD spent per patient on the Explore Transplant Ontario website^a^ • Mean (SD) of Explore Transplant Ontario page views per year (incl. 2017, 2018, and 2019) ^a^ • Number of website clicks for the Patient Resource Hub^a^ • Proportion of survey respondents who considered the patient education resources helpful^b^	• Number of locally developed patient education resources^a^ • Number of adaptations to patient education infrastructure^a^ • Proportion of survey respondents giving Explore Transplant Ontario videos or brochures to patients or family members^b^ • Proportion of patient education resources used with patients or family members^b^ • Frequency of patient education resources used . . . ○ prior to the COVID-19 pandemic (prior to March 2020)^b^ ○ during the first wave of the COVID-19 pandemic (March—May 2020)^b^
Education for healthcare providers	• Number of Core Transplant Curriculum Webinars offered^a^ • Number of Explore Transplant Ontario training sessions hosted^a^ • Number of contacts with education support personnel^a^	• Proportion of Core Transplant Curriculum Webinars attended^a^, b • Mean number of Core Transplant Curriculum page views per year per program (incl. 2018 and 2019) ^a^ • Proportion of training sessions attended^a^ • Number of additional support/training activities provided^a^ • Number of providers who accessed the Explore Transplant Ontario website^a^ • Mean time (minutes) (SD) spent per provider on the Explore Transplant Ontario website^a^ • Mean (SD) of Explore Transplant Ontario page views per year (incl. 2017, 2018, and 2019) ^a^ • Number of clicks for the Provider Resource Hub^a^ • Proportion of survey respondents who . . . • considered the Core Transplant Curriculum Webinars helpful^b^ • who attended a training session^b^ • who participated in an additional support/training activity^b^ • who considered the Explore Transplant Ontario resources helpful^b^ • who participated in a teleconference with the education support personnel^b^ • who participated in an in-person meeting with any of the education support personnel^b^ • who accessed the Provider Resource Hub^b^ • who considered the provider educational resources helpful^b^ • who watched any of the Explore Transplant Ontario videos^b^ • who read any of the Explore Transplant Ontario brochures^b^	• Number of locally developed provider education resources (as measured by the Education Task Force) ^a^ • Number of adaptations to provider education infrastructure (as measured by the Education Task Force) ^a^ • Proportion of provider education resources used^b^
**3. Access to Support: Transplant Ambassador Program (TAP)**			
	• Number of TAP training events held^a^ • Number of troubleshooting sessions with TAP ambassadors held^a^	• Number of TAP ambassadors that received TAP training^a^ • CKD program readiness for TAP (ie, readiness on a scale of 1-5) (as rated by TAP Ambassadors) ^a^ • Number of Transplant Ambassador Program (TAP) leads^a^ • Total Number of TAP ambassadors^a^	• Proportion of QI team meetings attended by a TAP lead or co-lead^a^ • Mean (SD) of TAP ambassador shifts completed per month^a^ • Mean (SD) of meaningful interactions of TAP ambassadors per month^a^ • Mean (SD) of how rewarding TAP ambassador shifts were (as measured on a 5-point Likert scale) ^a^
**4. Program-level performance monitoring**			
	• Number of Quarterly Performance Reports delivered to QI team^a^ • Mean (SD) Quarterly Performance Reports delivered per year^a^ • Number of Transplant Eligibility lists delivered^a^ • Mean (SD) Transplant Eligibility lists delivered per year^a^	• Mean of Quarterly Performance Reports reviewed by QI team^b^ • Mean (SD) ease of interpretation of Quarterly Performance Report^b^ • Mean (SD) survey respondents reporting utility of reports for understanding how many patients in the respective CKD Program took steps to receive a kidney transplant^b^ • Mean proportion of Transplant Eligibility lists reviewed by QI team^b^ • Mean (SD) ease of interpretation of Transplant Eligibility lists^b^ • Mean (SD) rating of Transplant eligibility lists utility^b^	• Mean (SD) of Quarterly Performance Report contribution to more patients being assessed for transplant eligibility^b^ • Proportion of survey respondents indicating Quarterly Performance Reports were shared outside of the QI team^b^ • Mean (SD) of healthcare providers outside of the QI team who reviewed the Quarterly Performance Report^b^ • Mean frequency (SD) with which healthcare providers outside of the QI team reviewed the Quarterly Performance Report^b^ • Mean (SD) of Quarterly Performance Report contribution to more patients being referred to transplant centres^b^ • Mean (SD) likelihood to continue using the Quarterly Performance Report^b^ • Mean (SD) Transplant Eligibility list perceived contribution to patients being assessed for transplant referral^b^ • Proportion of survey respondents indicating Transplant Eligibility list were shared outside of the QI team^b^ • Mean (SD) healthcare providers outside of the QI team who reviewed the Transplant Eligibility list^b^ • Mean frequency (SD) with which healthcare providers outside of the QI team reviewed the Transplant Eligibility list^b^ • Mean (SD) Transplant Eligibility list contribution to more patients being referred to transplant centres^b^ • Mean (SD) likelihood to continue using the Transplant Eligibility list^b^

*Note.* EnAKT LKD = Enhance Access to Kidney Transplantation and Living Kidney Donation; CKD = chronic kidney disease; QI = quality improvement; IDEAS = improving and driving excellence across sectors; TAP = transplant ambassador program; <plus others?>

a Data sourced from the trial implementation checklist.

b Data sourced from the online intervention group survey.

#### Data Collection

##### Online surveys

The survey completed by participants in the intervention group will assess components of intervention fidelity (delivery, receipt, and enactment) and the mechanism of change. Given that CKD programs in the usual-care group are not receiving the intervention, the usual-care group survey focuses on assessing barriers to kidney transplantation and living kidney donation, and contains questions to assess contamination (ie, whether the usual-care group was exposed to any of the intervention components) (Supplemental Appendices 2-3 contains the surveys for the intervention group and the control group, respectively).

To operationalize the NPT, we will use the Normalization Measure Development (NoMAD) instrument, a standardized set of questions tailored to a specific intervention; these questions will evaluate the extent to which the intervention was adopted by the healthcare team.^19^ Survey questions will assess how “normalized” the intervention has become (using a scale from 0 [not at all] to 10 [completely]), and will also include the 22-item NoMAD instrument to assess the 4 NPT constructs with responses measured on a 5-point Likert scale (strongly agree to strongly disagree, or not relevant).

Using the TDF, we will assess perceived barriers to kidney transplantation and living kidney donation from the perspective of healthcare staff in all 27 CKD programs. A broad list of barriers was identified from a patient-led workshop, from conversations with patients and healthcare providers, and from the literature.^20-23^ We have previously published this list of barriers to kidney transplantation and living donation.^11^ From this list, we identified which barriers are targeted by the intervention and we developed survey items to assess the extent to which respondents experienced 21 barriers to helping patients access kidney transplantation and/or living kidney donation (Supplemental Appendix 4). Respondents will be asked to consider potential barriers from 4 perspectives: (1) the healthcare provider (eg, I am not confident in my ability to promote kidney transplant and living donation), (2) the CKD program (eg, resources [including time] are not allocated to promoting transplant in our program), (3) patients with CKD (eg, patients lack access to educational materials about kidney transplantation and living kidney donation), and (4) living kidney donor candidates (eg, potential living kidney donors demonstrate a lack of knowledge about living kidney donation).

Respondents from CKD programs in the intervention and usual-care groups will be asked to recall which barriers they experienced at multiple time points, rating their level of agreement with the presence of each barrier at each time point using a 5-point Likert scale (strongly agree to strongly disagree). For CKD programs randomized to the intervention, respondents will be asked to rate the degree to which they experienced each barrier during 4 time periods: (1) experienced before the EnAKT LKD trial launched in their program (before Fall 2017), (2) experienced before the COVID-19 pandemic (Fall 2017 to March 2020), and (3) experienced now (survey completed between December 2020 and February 2021). The control group will respond to each of the following time intervals: (1) experienced before the COVID-19 pandemic (before March 2020), (2) experienced during the first wave of the COVID-19 pandemic (March to May 2020), and (3) experienced now (survey completed between December 2020 and February 2021). We will assess which barriers were experienced by respondents in the usual-care group to determine if there were differences in healthcare providers’ perception of barriers over time that were not related to the intervention. Additionally, to assess the potential for contamination in the usual-care group, the usual-care group survey will include a list of possible activities that could be used to help patients access kidney transplantation (Supplemental Appendix 3); respondents will be asked to indicate which of the activities were used during the trial period.

##### Semi-structured interviews

For the CKD programs receiving the intervention, we will conduct one-on-one semi-structured interviews with each quality improvement team lead and another healthcare professional. Interviews will be conducted by telephone or videoconference (Zoom or Microsoft Teams).

Interviews will include questions informed by NPT as well as open-ended questions to elicit participant reflection (Supplemental Appendix 4). Interviewees will also be asked about the intervention launch event, about their experience delivering each intervention component (including during the COVID-19 pandemic), and about any barriers or enablers encountered. They will also be asked how the intervention affected their work related to kidney transplantation and living kidney donation.

*Interviews will be audio-recorded and transcribed verbatim, verified by the interviewer (MW) and de-identified. Qualitative data will then be imported into NVivo 11 for analysis. To monitor the progress of the interviews and permit follow-up on issues that may emerge from the data, we will conduct interviewing, transcription, and analysis concurrently.*

##### Trial implementation checklist

We will review routine documentation describing the delivery and implementation of the EnAKT LKD trial at each CKD program as an additional method to assess fidelity of delivery, receipt, and enactment of the EnAKT LKD intervention (objectives 1-3). We will use the EnAKT LKD Logic Model and TIDieR checklist to collate a list of documents to request from the ORN-TGLN project team and CKD programs. Examples of these documents include participation logs of monthly collaborative calls, attendance at collaborative events, Explore Transplant Ontario website access, quality improvement team charters, and meeting agendas. In addition, the EnAKT LKD trial team collected specific process measures (eg, the number of patients who received ≥30 minutes of education) throughout the trial period. These data will also be integrated into our process data. If the EnAKT LKD trial shows an improvement in the primary outcome and the process evaluation shows that the intervention components were delivered, received, and enacted with high fidelity, and that the intervention addressed relevant barriers, we will have greater confidence that effects in the main trial can be attributed to the intervention.

#### Analysis plan

##### Quantitative data (surveys)

Quantitative survey data assessing the fidelity of the intervention delivery, receipt, and enactment (objectives 1-3) will be summarized as means (standard deviations [SD]), medians (25th, 75th percentiles), and proportions as described for each indicator in Table 1. Given the variable types of indicator data, it would not be meaningful to compute an overall fidelity score. Instead, we will seek to represent the range, across programs, of the degree to which each intervention component was delivered, received, and enacted with fidelity. These findings will be used in combination with main trial results to improve and streamline the intervention: If the intervention is shown to be effective, the fidelity data will help identify intervention components that had low or variable fidelity that could be improved or removed from future iterations of the intervention; if the intervention is not shown to be effective, the fidelity data may help identify which elements were not properly delivered, received, or enacted, and these elements could be modified in future iterations of the intervention.

For objective 4, quantitative survey data from NoMAD will be summarized using means and SDs for each of the 4 process-oriented NPT constructs. The internal consistency of items within each construct will be assessed using Cronbach’s alpha. Higher scores indicate greater integration of the intervention into the respondents’ workflow. Quantitative survey data on the TDF-derived list of barriers to kidney transplantation and living kidney donation (which were captured in surveys completed by the intervention group and the usual-care group) will be summarized using means and SDs. We will calculate the mean for the intervention group at 3 timepoints: before the intervention launched in November 2017, between November 2017 and March 2020 when the pandemic began, and currently (survey distributed between December 2020 and February 2021). The mean scores for the control group will be calculated at 3 timepoints: before the pandemic began in March 2020, during the first wave of the pandemic between March 2020 and May 2020, and currently (survey completed December 2020 to February 2021). Responses will be used to explore variation in barriers that occurred during the intervention period that were not related to the intervention.

##### Qualitative data (interviews with staff in the intervention-group)

Objective 4 will also be assessed through the interview portion that asks NPT-informed questions. Data (ie, interview transcripts and coded text) will be analyzed using directed content analysis guided by NPT.^24^ Data codes will be generated by labeling 1 to 2 lines of text with a descriptive label and then subsequently sorting these into the 4 NPT constructs. Codes representing similar thematic topics will be grouped into NPT sub-constructs; these will be defined and documented in a codebook. This coding will then inform a qualitative analysis using factors from NPT to understand the process of implementing and integrating (ie, enacting) the intervention at CKD programs. To verify the emerging analysis, a second analyst will review a preliminary set of themes to assess how well the data are represented and the relevance of data within codes and the associated constructs. Where differences in interpretation arise, the 2 analysts will discuss until agreement is reached and amendments will be made to the codebook as necessary.

## Timeline

The EnAKT LKD trial will be delivered to the intervention group until December 31, 2021. The primary outcome of the EnAKT LKD trial will be analyzed within approximately 1 year of the trial’s end date. The process evaluation surveys and interviews were completed in spring 2021, and the analysis is planned to begin in late 2021 after collecting all required data pertaining to the delivery and implementation of EnAKT LKD at each CKD program. The analysis of the process evaluation will occur before knowing the trial results with the 2 analyses being completed independently.

## Ethical Considerations

### Consent Process

This protocol has received approval from the Ottawa Hospital Research Institute Research Ethics Board (REB 20200426-01H). We will obtain informed consent from all individuals who agree to participate in the study. All participant recruitment and consent materials were reviewed and received ethics approval.

### Privacy

Only the research staff will have access to the collected data. Identifying information from surveys and interview transcripts will be removed before analysis. All electronic data will be password protected, encrypted, and stored in firewall-protected servers at the lead site. Any hardcopy records will be housed at the host center in locked storage facilities with controlled access in compliance with the Tri-Council Policy Statement on Ethical Conduct for Research Involving Humans and Good Clinical Practice Guidelines.^25^

## Discussion

The EnAKT LKD cluster-randomized trial is testing whether a quality improvement intervention increases patients’ access to kidney transplantation and living kidney donation. The intervention is complex, with several interacting components, and is being implemented as part of routine clinical care in 13 CKD programs in Ontario.

To help us interpret the trial findings, we designed a process evaluation following guidelines from the UK Medical Research Council framework for evaluating complex interventions. The evaluation will be conducted alongside the EnAKT LKD trial and use a mixed-methods approach to evaluate the fidelity of the intervention’s implementation and potential mechanisms of change. The results of the evaluation will provide insight into how and why the intervention is effective or ineffective. If the main trial does not demonstrate an improvement in the primary outcome, process evaluation findings will help to understand whether the intervention itself is flawed or whether its delivery was suboptimal and whether the intervention or its delivery can be optimized for CKD programs which adopt the intervention in the future.

In this process evaluation, we use 2 complementary implementation science theoretical frameworks (Theoretical Domains Framework and Normalization Process Theory) to explore the potential mechanism of action of the EnAKT LKD trial. The use of theoretical frameworks is novel in the kidney transplantation and living donation setting, and the combination of NPT and TDF is novel across the broader QI literature. By incorporating theoretical frameworks into our process evaluation, we will better understand the contextual factors that can affect the success of complex interventions designed to increase access to transplantation and living kidney donation. Furthermore, we add to the growing literature of theory-based, mixed-methods process evaluations of complex interventions.^14^

The proposed study has several strengths. We published this protocol and are conducting the evaluation in advance of knowing the trial results to improve the quality of our evaluation process. We will use a mixed-methods approach and obtain process data from various sources including review of trial implementation documents, surveys, and interviews. The design follows the EnAKT LKD logic model and uses strong theoretical frameworks which are increasingly used to guide the evaluation of complex interventions in the healthcare setting.

There are limitations and challenges anticipated for the proposed process evaluation. First, there is no baseline data collection and participants are asked to retrospectively consider the barriers and enablers they experienced to increasing patients’ access to kidney transplantation and living kidney donation before the delivery of the EnAKT LKD trial. Second, the trial period spans several years, and participants may suffer from poor recall. To reduce this potential bias, the recall periods for survey and interview questions are limited to less than 1 year and wherever possible the study team provides image cues to facilitate recollection. Third, the COVID-19 pandemic essentially halted all transplant and donation activity across the province (both deceased and living donor kidney transplants), and therefore all elements of fidelity (delivery, receipt, and enactment) are likely to be impacted by the pandemic. To understand better the extent to which the pandemic affected intervention activities, the survey and interviews include questions about how the EnAKT LKD intervention components were treated during the pandemic period. Finally, since we only surveyed and interviewed healthcare providers, we lack information about how the intervention components were delivered to patients and whether they perceived the intervention to be effective or not.

## Conclusion

This protocol describes a mixed-methods, theory-based process evaluation conducted alongside the EnAKT LKD randomized trial that is testing a complex intervention designed to increase patient access to kidney transplantation and living kidney donation. The results of this process evaluation will aid in interpretation of trial outcomes and help optimize the intervention for successful implementation at other CKD programs.

## Supplemental Material

sj-docx-1-cjk-10.1177_20543581221084502 – Supplemental material for Protocol for a Process Evaluation of the Quality Improvement Intervention to Enhance Access to Kidney Transplantation and Living Kidney Donation (EnAKT LKD) Cluster-Randomized Clinical Trial Click here for additional data file. Supplemental material, sj-docx-1-cjk-10.1177_20543581221084502 for Protocol for a Process Evaluation of the Quality Improvement Intervention to Enhance Access to Kidney Transplantation and Living Kidney Donation (EnAKT LKD) Cluster-Randomized Clinical Trial by Seychelle Yohanna, Mackenzie Wilson, Kyla L. Naylor, Amit X. Garg, Jessica M. Sontrop, Dmitri Belenko, Lori Elliott, Susan McKenzie, Sara Macanovic, Istvan Mucsi, Rachel Patzer, Irina Voronin, Iris Lui, Peter G. Blake, Amy D. Waterman, Darin Treleaven and Justin Presseau in Canadian Journal of Kidney Health and Disease
