# Supplementary material for: Protocol for a Process Evaluation of the Quality Improvement Intervention to Enhance Access to Kidney Transplantation and Living Kidney Donation (EnAKT LKD) Cluster-Randomized Clinical Trial
Source: Can J Kidney Health Dis. 2022 Mar 19;9:20543581221084502. doi: 10.1177/20543581221084502 (PMC8943297; doi:10.1177/20543581221084502)
Supplement: sj-docx-1-cjk-10.1177_20543581221084502 – Supplemental material for Protocol for a Process Evaluation of the Quality Improvement Intervention to Enhance Access to Kidney Transplantation and Living Kidney Donation (EnAKT LKD) Cluster-Randomized Clinical Trial [file sj-docx-1-cjk-10.1177_20543581221084502.docx]

***Supplemental Appendix 1.*** TiDier Checklist.

EnAKT LKD intervention components as per the Template for Intervention Description and Replication Criteria (TiDIER)

| **Brief name** | **Quality Improvement Intervention to Enhance Access to Kidney Transplantation and Living Kidney Donation (EnAKT LKD)** | | | | **Usual Care** |
| --- | --- | --- | --- | --- | --- |
| **Components** | **Local quality improvement teams and administrative support** | **Education for providers, patients and families** | **Transplant Ambassador Program (TAP)** | **Program-level performance reports and oversight by program leaders** | **Usual Care** |
| **Subcomponents** | -Local quality improvement (QI)* leads  -Administrative support from ORN-TGLN staff  -Financial support to CKD programs | -Patient and provider online transplant resource hub  -Explore Transplant Ontario  -Ontario Core Transplant Curriculum  -Social Networking (e.g., encouraged transplant education events for staff, patients and families) | -Support from kidney transplant recipients and living kidney donors  -Transplant education  -Aid in navigating the transplant process | -Leverage existing administrative healthcare data and data sharing  -Quarterly performance reports on key transplant metrics  -Transplant eligibility lists for CKD programs | -Support access to kidney transplantation and living kidney donation as usual. |
| **Why** | -Improving healthcare processes is a team effort, so each CKD program has a QI team to take charge of improvement efforts and ensures key stakeholders are involved in the implementation of the intervention. Additionally, creating a “local” QI team enables and supports grass roots changes, from individuals who can best appreciate their local context.    -Some intervention activities require provincial coordination (e.g., data component required a data sharing agreement between ORN and TGLN). CKD program staff are busy; administrative support form ORN and TGLN will help support the intervention implementation and other local initiatives. | -The need for more effective transplant-related education was identified by a patient-led roundtable and confirmed in a needs assessment conducted by the education task group.  -Early transplant education has been shown to increase referral rates and decrease disparities in access to transplantation^26–35^ | -Support was identified as a key priority for patients. Peer mentors can provide both practical advice and emotional support, inspire family and friends to consider living kidney donation, and motivate patients to complete essential steps on the transplant pathway. They can often spend more time than healthcare providers discussing transplantation with patients and families | -A barrier to improving transplant performance that CKD programs identified is the lack of high-quality transplant related performance data.  -Transplant eligibility lists serve as a notification to remind a CKD program to consider patients who appear to be eligible for transplant but have not been referred. This helps avoid patients getting lost in the system. | -CKD program healthcare providers have not traditionally placed a heavy emphasis on access to transplant in their day-to-day work with many competing priorities (e.g., improving home dialysis starts), limited time, and limited transplant specific training. |
| **What materials** | -Your CKD Transplant Quality Improvement Team document outlined the key stakeholders that should be selected for the QI team.  -Team charter included instructions on how to formulate appropriate aim statements and provided an example team charter. Charter was then sent to ORN-TGLN for review.  -QI workshop provided teams with the framework and tools they need to enact change at their program. QI teams could access to online content for future reference. Teams were provided with instructions on how to complete a “plan-do-study-act” cycle. | -Materials and methods to develop a strategy to improve kidney transplant education to best suit CKD local practice provided. Programs were encouraged to develop additional educational resources, optimize patient education infrastructure, and disseminate the EnAKT LKD education initiative to staff and patients.  -Education for healthcare providers included 1) educational toolkit ([renalnetwork.on.ca/TransplantProviderHub](file:///C:\Users\seychelleyohanna\Desktop\Desktop%20-%20Seychelle’s%20MacBook%20Pro\Desktop\renalnetwork.on.ca\TransplantProviderHub)); 2) The Ontario Core Transplant Curriculum; and 3) Explore Transplant Ontario training ([etontario.org](https://etontario.org/))  -Education for patients and families (which includes potential donors) included 1) an educational toolkit ([renalnetwork.on.ca/TransplantPatientHub](file:///C:\Users\seychelleyohanna\Desktop\Desktop%20-%20Seychelle’s%20MacBook%20Pro\Desktop\renalnetwork.on.ca\TransplantPatientHub)); 2) Explore Transplant [etontario.org](https://etontario.org/); and 3) Social networking (CKD programs were encouraged to host workshops and social networking events such as educational movie nights) | -TAP guide provides a reference tool for ambassadors and CKD programs to understand the requirements to participate and the role and responsibility of the ambassador. Outlines the mission and goals of the peer mentorship program, how to recruit new members, training for new members, etc.  -TAP data collection sheet recording information on each interaction with a patient or family member.  -TAP website provides a centralized location for recruitment, support and resources for ambassadors. Provides resources for patients and allows interested patients to contact an ambassador. (<https://transplantambassadors.ca/>)  -TAP toll-free number allows interested patients and families to call a number to connect with an ambassador.  -TAP posters for CKD programs promoting the TAP program to patients and families. | -Data sharing agreement between ORN-TGLN  -Quarterly performance reports delivered to QI teams.  -Transplant eligibility lists generated for each CKD program’s QI team | QI teams:  -Not encouraged to form local quality improvement teams to improve local performance  Education:  -CKD program education and materials vary across programs. No dedicated transplant resource hub.  Peer support:  -No established transplant-specific support program. The kidney foundation of Canada has a peer support program that is a resource for all people in the province to use. This support program is not integrated into care and is available for any aspect of kidney disease (not specific to transplant).  Data:  -No quarterly performance reports or transplant eligibility lists provided. |
| **What procedures** | -QI teams appointed a champion (team lead)  -Teams participated in a 2-day QI workshop.  -Teams were asked to use process mapping to review their transplant referral and education pathways.  -Local QI team encouraged to meet regularly during the trial to discuss, review, and develop a plan to improve performance  -All 13 CKD program QI teams meet together monthly via teleconference or in-person to share progress and to discuss strategies for overcoming barriers  -Regular completion of Plan-Do-Study-Act (PDSA) cycles  -Approximately three full-time equivalent positions from the ORN and TGLN (a business strategist, a senior analyst, an analyst, project manager)  -Each CKD program allocated $10,000 per year to support intervention implementation and other local initiatives  -18-member panel meets throughout the intervention to discuss challenges and success of the strategy and ideas for how to improve the strategy.  -In-person visits from the provincial medical lead in transplantation occurred as needed | -Implemented by the EnAKT LKD education team (comprised of three transplant education experts) in collaboration with each CKD program’s quality improvement team.  -Additional support from the education team was provided throughout the trial, as needed. | -TAP training to ambassadors by TAP coordinators to provide ambassadors with the skills to provide effective support.  -Appointing a lead and co-lead ambassador for each CKD program.  -Transplant ambassadors were members of the QI teams: ensuring the patient and donor perspective was represented on the QI team. Also, the QI team members and the TAP lead and co-lead worked closely together to ensure the TAP program success. The QI team would support ambassadors with resources (if needed) and help them trouble shoot local issues.  -Troubleshooting sessions for ambassadors at in-person meetings and monthly calls with ORN-TGLN.  -Regular teleconference meetings with all TAP members to discuss various issues. Examples include recruitment, strategies to integrate into dialysis units and multi-care kidney clinics, how to communicate with patients and families.  -Ambassador phone and email support from TAP coordinators  -Transplant ambassadors collected data about each interaction with a patient or family member. Data collection (e.g., number of meaningful patient interactions) helped to offset the data collection being done by other QI members (e.g., number of patients who completed ≥30 minutes of education).  **-**Ambassadors are visible (bright green vest with TAP logo and a large-print invitation to “Ask me about Kidney Transplantation”) in multi-care kidney clinics and dialysis units for patients and families to approach  -CKD program healthcare providers are able to identify patients who would benefit from being approached by an ambassador  -Ambassadors can provide personal contact information for patients and families to arrange future meetings | -Quarterly performance report contains process and outcome metrics in kidney transplant. Reviewed by QI teams and in accountability meetings with the ORN-TGLN. QI teams were also encouraged to share local data with all relevant healthcare providers and stakeholders in their program.  - QI teams encouraged to share eligibility lists with staff in their peritoneal dialysis programs, hemodialysis programs, and multi-care kidney clinics.  -Best practices in audit and feedback were utilized for reports. | -Delivery of transplant education is highly variable. Most CKD programs employ an educator (usually a nurse) who dedicates varying amounts of time providing modality education to patients. Modality education is usually provided at the nephrologist request and is a one-time, comprehensive session that explores all options for kidney replacement therapy (e.g., dialysis options, kidney transplantation).  -No additional financial support to CKD program provided. |
| **Who provided** | -All documents and support were provided by ORN- TGLN administration who have expertise in the field of access to transplantation. QI team documents were also created in collaboration with a provincial expert in quality improvement and transplantation (S Yohanna).  -Letter to transplant administrators provided by the Vice President of Trillium Gift of Life Network  -2-day QI workshop was provided by IDEAS (Improving and Driving Excellence Across Sectors) Quality Improvement training program (<https://www.ideasontario.ca/>).  -Financial support ($10,000 per year to CKD programs) | -Ontario Core Curriculum: Various transplant providers provided the webinars  -Explore Transplant Ontario (ETO) Training: Dr. Waterman (program creator).  -After initial training provided by Dr. Waterman, she was also available for additional in-person visits or teleconferences.  -ETO materials distributed by the ORN-TGLN  -Education support teleconferences: EnAKT LKD education team  -Educational toolkit for providers: EnAKT LKD education team complied transplant education material online and developed new educational materials and made them available online.  -Education toolkit for patients and families (which include potential donors): EnAKT LKD education team complied material online and developed new educational materials and made them available online. Healthcare providers and transplant ambassadors, or volunteers could distribute materials to patients.  -Explore Transplant Ontario materials were offered to patients by frontline healthcare providers, transplant ambassadors and volunteers.  -Social networking - local CKD programs developed and hosted. | -Transplant Ambassador Program Guide for ambassadors: TAP creators  -TAP data collection sheet: TAP coordinators  -TAP website: TAP creators  -TAP toll free number: TAP coordinators/ORN-TGLN  -Appointing a lead and co-lead ambassador for each CKD program: ORN-TGLN provided guidance on QI team members and the TAP creators helped individual teams identify leads  -Transplant ambassador lead and co-leads were members of the QI teams: guidance from ORN-TGLN  -Transplant ambassadors collected data about each interaction with a patient or family member: ambassadors  -TAP Posters: TAP creators | -Reports were provided by the ORN | -CKD program staff (e.g., nurses, nephrologists) will continue to provide usual care to patients. Administrative support from the ORN-TGLN is not regularly provided. Support is only provided if a specific challenge has been identified at a program. |
| **How (method of delivery)** | -All materials were sent via email unless otherwise indicated.  - QI workshop was delivered in-person  -Local QI teams encouraged to meet in-person (prior to the COVID-19 pandemic)  -All 13 CKD program QI teams meet monthly via teleconference or in-person  -ORN-TGLN project team teleconference (18-member panel) | -Educational toolkits for providers: Website  -Educational toolkit for patients and families: website  -Ontario Core Transplant Curriculum: webinars (webinars were archived when a healthcare professional could not make the event).  -Explore Transplant Ontario Training: in-person workshop  -Education support teleconferences: telephone conference  -Living kidney donation expert workshop: in-person workshop  -Explore Transplant Ontario: DVD, website, pamphlets | -Ambassadors were visible and available in the multi-care kidney clinics and dialysis unit to meet and talk to patients in person.  -Ambassadors were available to connect with patients by email or telephone.  -In some cases, healthcare providers helped identify patients who might benefit from connecting with an ambassador. | -Quarterly reports distributed to QI team lead via secure file transfer.  -Eligibility lists utilized a secure server where teams provided a username and password for access. |  |
| Where | -Local QI teams undertook QI activities at their home CKD program  -QI workshop was provided in Toronto, Canada.  -ORN-TGLN administration located in Toronto, Canada | **-**Educational Toolkit for healthcare providers: CKD programs  -Ontario Core Transplant Curriculum: CKD programs and transplant centres  -Explore Transplant Ontario Training: occurred at an in-person event and delivered to CKD program staff and transplant ambassadors  -Education support teleconferences: CKD programs  -Living kidney donation expert workshop: In-person event held in Toronto, Canada  **-**Educational Toolkit for patients and families: CKD programs  -Explore Transplant Ontario: CKD programs  -Social Networking: CKD programs | -Dialysis units, multi-care kidney clinics or any place agreed upon by the patient and ambassador to meet. | -Created by ORN-TGLN and then distributed to each CKD program via secure file transfer. | -Care provided to CKD patients at the CKD programs (multi-care kidney clinics, dialysis units) by CKD staff (e.g., nurse, nephrologist). |
| When and how much | -Letter to transplant program administrators highlighting the strategy to increase patient access to transplantation and living kidney donation, local CKD process mapping of education and referral pathways, and instructions on how to create a QI team circulated prior to start of trial.  -QI collaborative meeting occurred once prior to the start of the trial and was a two-day session.  -Local QI team was encouraged to meet regularly to discuss, review and develop a plan to improve performance  -QI teams created a project charter with input from the ORN  -QI teams encouraged to regularly completed PDSA cycles and update team charters.  - All 13 CKD program QI teams meet monthly (collaborative conference calls)  -ORN-TGLN project team met regularly.  - Approximately 3 full time equivalent positions from ORN-TGLN throughout the trial.  -$10,000 provided yearly to CKD programs | **-**Ontario Core Transplant Curriculum: Several times throughout the trial  -Explore Transplant Ontario Training: Provided once before the trial started. Dr. Waterman was also available to provide additional in-site visits to help the programs and to provide teleconferences for support.  Education support teleconferences: As needed  -Living kidney donation expert workshop: In-person event held in the first year of the trial  -Educational Toolkit for healthcare professional: created at the beginning of the trial, new infographics were added as they were finalized  **-**Educational Toolkit for patients and families: created at the beginning of the trial, new infographics were added as they were finalized  -Explore Transplant Ontario: available throughout the trial.  -Social Networking: Throughout the trial as much as the CKD programs wanted. | -Highly variable according to transplant ambassador recruitment and availability, patient, and healthcare professional engagement. | -Program-level reports delivered every quarter throughout the trial. | -Not applicable |
| Tailoring | -Local QI team: Despite being given instruction about the appropriate team members, teams could still tailor how they created the QI team to suit their program. We recommended that they meet regularly but ultimately, they decided how often to meet with their team.  -Team charter: in developing their team charters, the QI teams were given the freedom to choose any area of transplant activity to focus on for improvement. They also were not required to show evidence that they were referring to, editing, utilizing this document after the outset of the trial.  -QI workshop: no tailoring  -Process mapping: teams were provided a process mapping template but were not required to use it.  -Monthly calls with 13 CKD program QI teams: strongly encouraged to participate on monthly phone calls but it was not an absolute requirement.  -Frequency of all meetings may be modified depending on participant availability and holidays. | -Ontario Core Transplant Curriculum: no tailoring  -Explore Transplant Ontario Training: no tailoring  -Education support teleconferences: CKD programs could request as needed  -Living kidney donation expert workshop: no tailoring  -Educational Toolkit for healthcare providers, patients, and families: no tailoring to material  -Social Networking: CKD programs could decide what events they would like to host/if they would like to host an event.  - It was at the discretion of the CKD program staff to attend the Core Transplant Curriculum webinars and to decide if/the amount of education a patient receives | -Recruitment strategies and conversations will vary among transplant ambassadors and CKD programs. | -No tailoring possible for quarterly reports.  -No tailoring possible for transplant eligibility lists. | -Variability in the amount of education delivered across CKD programs, with no standard for the duration or content of transplant education.  -Usual care group was not prevented from creating peer support program or quality improvement teams, but they were not given formal resources or supports to do so. |
| **How well (Planned)** | -ORN took QI lead attendance at monthly calls with 13 CKD programs.  -Agendas from local QI teams were collected from ORN. | -Collected measures on “Number of patients who completed Explore Transplant Ontario” and “Number of patients who received ≥ 30 mins of transplant education”. This information was provided in the quarterly performance reports given to CKD programs.  -The number of website visits to Explore Transplant Ontario were tracked and the attendance to Core Transplant Curriculum webinars. | -Collected measures on “Number of meaningful interactions between patients with kidney failure and transplant ambassadors” and  -“Number of meaningful interactions between living kidney donor candidates and transplant ambassadors”. This information was provided in the performance reports given to CKD programs. | -CKD programs who did not provide information on some of the process measures (e.g., number of patients who received ≥ 30 mins of transplant education) were encouraged by ORN to provide this information. | -A survey will be sent to the usual care group to understand activities they used to help their patients access kidney transplantation during the trial period. |

*QI teams include a champion (the team lead), an executive sponsor (usually a member of hospital administration), local personnel with quality improvement experience, a clinical leader, and at least two patients, at least one of whom is also involved in TAP.

**Abbreviations**: CKD, chronic kidney disease; ORN, Ontario Renal Network; PDSA, Plan-Do-Study-Act; QI, quality improvement; TGLN, Trillium Gift of Life Network

***Supplemental Appendix 2.*** Intervention group surveys.

***Supplemental Appendix 3.*** Control group survey.

***Supplemental Appendix 4.*** Barriers targeted by the EnAKT LKD strategy

| **Potential barriers addressed by the EnAKT LKD strategy** | |
| --- | --- |
| **Theoretical Domains**^†^ | **Provider-related barriers** |
| *Beliefs about capabilities* | I am not confident in my ability to promote kidney transplant and living donation |
| *Knowledge* | I am not sure which patients are eligible for transplant* |
| *Knowledge/Behavioural regulation* | I am not sure when to refer a patient to a transplant centre* |
| *Knowledge* | I am not sure what the process is for referring eligible patients for transplant at my program |
| *Knowledge* | I don’t know enough about living kidney donation to promote it to my patients |
| *Skills/Emotion* | I am not comfortable enough talking about living kidney donation with my patients |
| *Skills* | I am not sure how to help my patients find a living kidney donor |
| *Knowledge/Behavioural regulation* | I am not sure which patients would benefit from a living donor kidney transplant versus a deceased donor kidney transplant* |
|  | **Chronic Kidney Disease Program-related barriers** |
| *Social and professional role/Motivation and goals* | Transplant is not a priority in our program |
| *Environmental context/resources* | Resources (including time) are not allocated to promoting transplant in our program |
| *Environmental context/resources* | I am not provided with effective educational resources to promote kidney transplant |
| *Social influences* | There is insufficient communication with transplant centres to help move my patients through the evaluation process |
| *Knowledge/Behavioural regulation* | I do not have access to the necessary data to understand how well patients in our program are completing steps towards receiving a kidney transplant |
| *Knowledge/Behavioural regulation* | I do not have access to the necessary information to know if a patient is potentially eligible to receive a kidney transplant |
|  | **Patient-related barriers** |
| *Environmental context/resources* | Patients do not have access to educational materials about kidney transplantation and living kidney donation |
| *Social influences* | Patients do not demonstrate enough knowledge about transplant |
| *Social influences* | Patients demonstrate misconceptions about their eligibility for transplant |
| *Environmental context/resources* | Patients are not provided with transplant education that includes their family/support system |
| *Social influences* | Patients indicate the absence of interactions with past recipients and living kidney donors to understand the transplant process from a patient perspective* |
|  | **Living donor-related barriers** |
| *Social influences* | Potential living donors demonstrate a lack of knowledge about living kidney donation |
| *Social influences* | Potential living donors indicate a lack of peer support to help them through the living donor evaluation process |

* Key barriers targeted by the intervention identified by the research team for additional analysis

^†^ Theoretical domains are identified from the perspective of the healthcare provider

***Supplemental Appendix V.*** Intervention group interview guides.
